# Emergent canine visceral leishmaniasis in Argentina: Comparative diagnostics and relevance to proliferation of human disease

**DOI:** 10.1371/journal.pntd.0009552

**Published:** 2021-07-19

**Authors:** Kyoko Fujisawa, Charlotte Silcott-Niles, Poppy Simonson, Daniela Lamattina, Cristian A. Humeres, Tapan Bhattacharyya, Pascal Mertens, Caroline Thunissen, Victoria O’Rourke, Magdalena Pańczuk, James A. Whitworth, Oscar Daniel Salomón, Michael A. Miles

**Affiliations:** 1 Faculty of Infectious and Tropical Diseases, London School of Hygiene and Tropical Medicine, London, United Kingdom; 2 Instituto Nacional de Medicina Tropical (INMeT), ANLIS, Ministerio de Salud de la Nación, Puerto Iguazú, Misiones, Argentina; 3 Consejo Nacional de Investigaciones Científicas y Técnicas (CONICET), Puerto Iguazú, Misiones, Argentina; 4 Coris BioConcept, Gembloux, Belgium; University of Liverpool, UNITED KINGDOM

## Abstract

**Background:**

Visceral leishmaniasis (VL) is a zoonotic protozoal vector-borne disease that is a major public health challenge. In Argentina, canine (CVL) and human visceral leishmaniasis (HVL) have recently emerged. There is a lack of standardised diagnostic tests for CVL, which hinders control of CVL and HVL.

**Methodology/Principal findings:**

Sampling was carried out in Puerto Iguazú, Argentina, comprising 190 asymptomatic, oligosymptomatic and polysymptomatic dogs. The following diagnostics were applied: microscopy of lymph node aspirate (LNA); three immunochromatographic rapid diagnostic tests (RDTs), prototype rK28-ICT, rK39-ICT (both Coris BioConcept), commercial rK39 (InBios); ELISA for IgG, IgG1 and IgG2, against rK28, rK39 or crude lysate antigen. DNA detection and analysis, with 30 dogs, was of the ITS1 region using skin samples, and loop-mediated isothermal amplification (LAMP; Eiken Loopamp) of buffy coat, skin scrape or LNA. 15.4% of dogs were positive by LNA microscopy. The rK28 RDT had higher seropositivity rate (61%) than either a prototype rK39 RDT (31.4%) or commercial rK39 RDT (18.8%), without cross-reactivity with six other pathogens. IgG anti-rK39 ELISA antibody titres, but not IgG2, were positively correlated with number of clinical signs. LAMP with LNA had a higher positivity rate than PCR; buffy coat sampling was more sensitive than skin scrape. ITS1 confirmed *Leishmania* (*Leishmania*) *infantum* as the agent of CVL. *Leishmania* (*Viannia*) spp. was detected in skin samples from two dogs, compatible with *Leishmania* (*Viannia*) *braziliensis*.

**Conclusions/Significance:**

Seroprevalence confirmed rapid increase in CVL in Puerto Iguazú. The rK28 RDT test potentially has great value for improved point-of-care diagnosis. Given cost reduction and accessibility, commercial LAMP may be applicable to buffy coat. RDT biomarkers of CVL clinical status are required to combat spread of CVL and HVL. The presence of *Viannia*, perhaps as an agent of human mucocutaneous leishmaniasis (MCL), highlights the need for vigilance and surveillance.

## Author summary

Visceral leishmaniasis (VL) is a widespread parasitic disease caused by sand fly-borne parasites of the *Leishmania donovani* complex (*L*. *donovani* and *L*. *infantum*). Without early diagnosis and successful chemotherapy symptomatic human VL is fatal. Dogs are reservoir hosts of *L*. *infantum*, and canine visceral leishmaniasis (CVL) often precedes outbreaks in local human populations. With dogs sampled within a region of emergent CVL in northern Argentina, we compared a range of diagnostic techniques, including microscopy, serology by enzyme linked immunosorbent assays (ELISAs) and rapid diagnostic tests (RDTs), and detection and identification of *Leishmania* DNA. Novel serological assays based on the rK28 antigen were more sensitive than those based on rK39, and without evidence of cross reaction with six other canine pathogens. A commercial DNA detection kit (LAMP; Eiken Loopamp), used for the first time with CVL, was more sensitive than PCR on lymph node samples. Unexpectedly, we also found canine infection with the *Viannia* sub-genus of *Leishmania*. Our results reinforce the need for improved diagnosis, vigilance, surveillance and control of CVL.

## Introduction

The leishmaniases are widespread sand fly transmitted neglected infectious diseases (NTDs) [1]. *Leishmania* (*Leishmania*) *donovani* is the predominant cause of human visceral leishmaniasis (HVL) in Asia and Africa, where transmission is largely anthroponotic. In contrast, HVL due to *Leishmania* (*Leishmania*) *infantum* in Latin America and the Mediterranean region, has canine visceral leishmaniasis (CVL) as a highly effective reservoir. In Latin America, one of the several agents of human cutaneous leishmaniasis (HCL), zoonotic *Leishmania* (*Viannia*) *braziliensis*, is associated with destructive metastatic mucocutaneous leishmaniasis (MCL) [2,3]

There is an increasing threat of spread of the leishmaniases to new regions, due to factors such as climate change, movement of human and reservoir populations, urbanisation and deepening of social inequalities [4].

Canine visceral leishmaniasis is endemic amongst dogs in many countries worldwide [5]. Although the transmission is principally via the sand fly vector, occasional direct and vertical transmission between dogs has been reported [6,7]. There is a broad spectrum of CVL clinical presentations after infection, from asymptomatic (up to 80% of infected dogs in some locations) to fatal systemic disease [8]. The most common clinical signs are systemic (generalised lymphadenopathy, weight loss, lethargy) and cutaneous (dermatitis, alopecia, onychogryphosis) [9].

Because the spread of HVL follows that of CVL [10], the diagnosis of infected dogs is vital for public health as well as canine health and welfare. A variety of diagnostic methods is used for CVL, both serological and molecular, positive tissue aspirate microscopy being the gold standard [11]. There is no curative chemotherapy for dogs [12], and no canine vaccine that is proven to have a positive public health outcome [13]. Diagnosis is generally used to inform selective culling of infected dogs in areas where HVL is a public health concern [14], which can reduce HVL incidence [15]. Although there has been some recent progress in the field of diagnosis of CVL, such as the development of rapid diagnostic tests (RDTs) based on the *Leishmania* rK28 or rK39 antigens, there remain multiple challenges in CVL diagnosis. Stray dogs form a large part of the canine reservoir in endemic areas and these are difficult to trace, meaning losses to follow up are common and repeated surveillance is difficult. Point of care RDTs that meet the ASSURED criteria (Affordable, Sensitive, Specific, User-Friendly, Rapid and robust, Equipment-free and Deliverable) are therefore desirable [16]. The extremely variable clinical presentation means identification of ‘super-spreader’ dogs that are most infectious to sand flies is difficult, particularly for those dogs that are asymptomatic. There is currently no test that can definitively diagnose infected asymptomatic dogs. Furthermore, if a potentially efficacious CVL vaccine becomes widely available, an RDT will be required to differentiate vaccine-induced immune response from natural infection [17]. Other current restrictions that hamper disease control include time delays, cost, and test limitations, in particular low sensitivity meaning many positive dogs are not identified.

Canine and human visceral leishmaniasis are emerging diseases in the Iguazú department and within Puerto Iguazú city, of Misiones Province, Argentina, near the triple border with Brazil to the north and Paraguay to the west. Human cutaneous leishmaniasis (HCL) is endemic in the north of the country, caused by *Leishmania* (*Leishmania*) *amazonensis*, *L*. *(V*.*) braziliensis and Leishmania (Viannia) guyanensis* [18]. However HVL, caused by *L*. *infantum*, is a newly emerging disease in Argentina [18]. The country’s first non-imported HVL case occurred in Posadas, Misiones Province, in 2006, in association with cases of *L*. *infantum* CVL in dogs [19]. In Puerto Iguazú human and canine infection with *L*. *infantum* was first confirmed in 2014 [20]. The location suggests that the vector (*Lutzomyia longipalpis*) and parasite have spread from neighbouring Brazil and Paraguay [21]. A recent study found prevalence rates of 26.2% in 2014 and 17.5% in 2018 amongst dogs in Puerto Iguazú [21]. As CVL is newly emerging in Argentina, there has been little exploration of the available diagnostic tests in the country, although a recent study compared RDTs for CVL in Oberá city, Misiones Province, Argentina [22].

The cross-sectional study described here compares a variety of established and novel tests for CVL, with a focus on their potential application to control the disease in Argentina. Improved diagnoses, with molecular identification of the disease agents, are key to control both canine and human leishmaniases. More sensitive and specific identification of CVL has a measurable public health impact via control of the reservoir host and thus by reduction of incidence and spread of HVL.

## Methods

### Ethics statement

The research was approved by the London School of Hygiene and Tropical Medicine (LSHTM) Ethics Committee, and the Ethics Committee in Clinical Investigation at the Ministry of Health, Argentina. Formal verbal consent was obtained from each dog owner before the clinical examination and sampling.

### Study location

**Fig 1:**
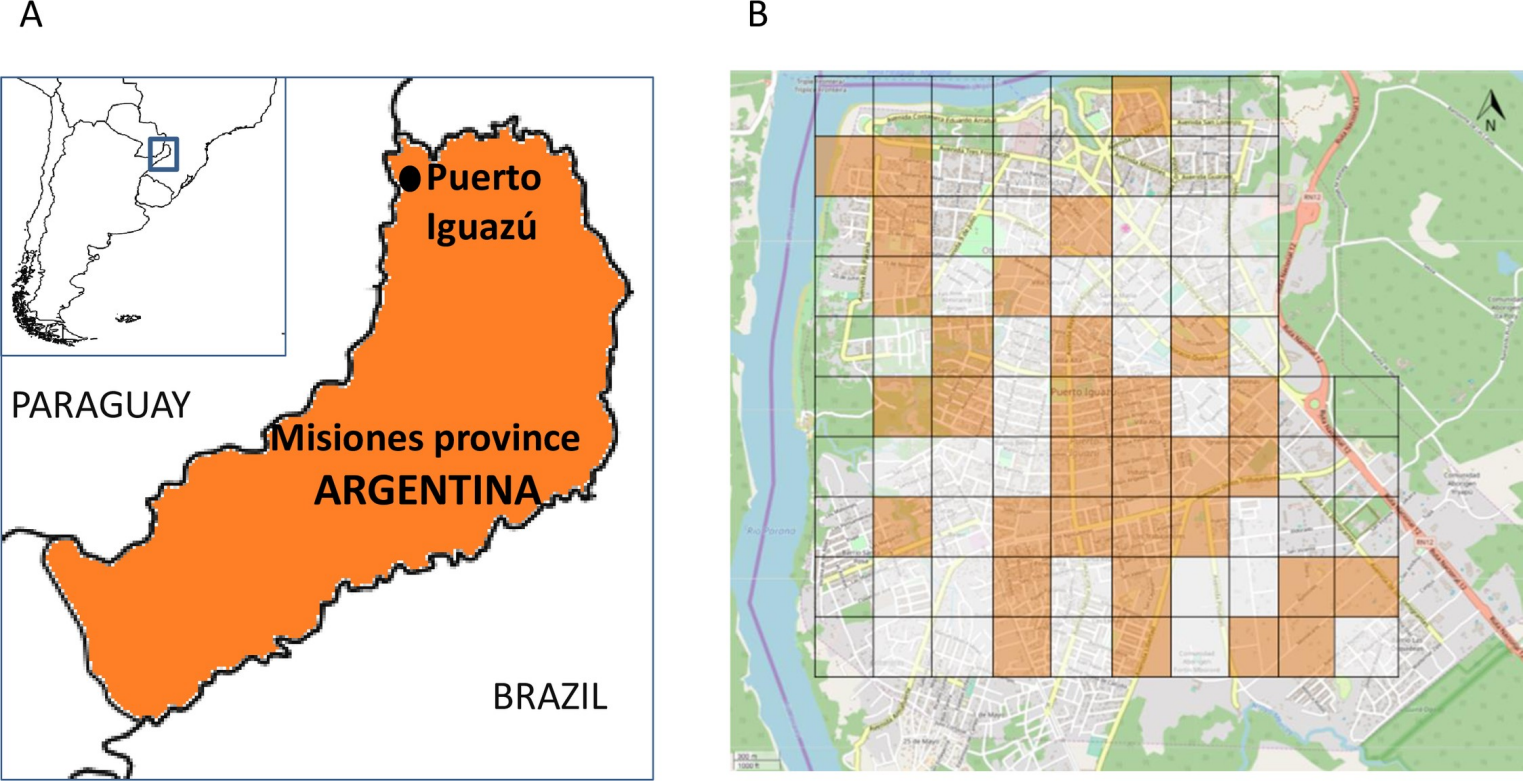
Study setting. A) Puerto Iguazú, Misiones province, Argentina, in the border region with Brazil and Paraguay, B) Puerto Iguazú city; blocks in orange show the 32 selected study sites. Map sources: for Fig 1A, https://www.simplemappr.net/; for Fig 1B, https://www.openstreetmap.org/.

The study was carried out in the city of Puerto Iguazú, Misiones Province, Argentina, in July and August 2018, with additional sampling carried out in June and July 2019. Puerto Iguazú (population 42,849 circa 2010 [23]) is located near the triple border with Paraguay and Brazil, and has a subtropical climate, with no dry season (Fig 1). The Iguazú National Park of subtropical Atlantic Forest (67,698 hectares) borders the west of the municipality.

### Sample collection

After dividing the city into an 80-cell grid of 400 m^2^ (Fig 1), 32 blocks were randomly selected for sampling [21]. Within each block, the domicile with the highest predicted risk was chosen using worst-scenario sampling (critical site criteria being presence of thick vegetation, high humidity, shadow, high proportion of organic matter on the soil from fruit trees and/or animal faeces, and bloodmeal sources from chickens and dogs) [21,24]. Up to five dogs living in or near the selected domicile were sampled; this resulted in a total sample size of 160 dogs from 77 households.

The dog owners were asked about the dog’s characteristics (age, sex and breed). Each dog was examined for clinical signs of CVL–lymphadenopathy, onychogryphosis, chancre, dermatitis, weight loss, conjunctivitis, and localised or generalised alopecia–and categorised as asymptomatic, oligosymptomatic (one or two symptoms), or polysymptomatic (three or more symptoms).

A blood sample, fine needle aspiration of the popliteal lymph node, saliva sample, and skin scrape were taken from each dog as follows. Two millilitres of peripheral blood were collected from the cephalic vein into non-anticoagulant tubes and EDTA tubes. Serum was separated from the blood in non-anticoagulant tubes after centrifugation. Buffy coat was isolated from the EDTA whole blood by centrifugation with Ficoll-Paque PLUS density gradient medium (GE Healthcare, Sweden). Lymph node aspiration (LNA) samples were used to prepare smears and were also suspended in 0.2 ml of phosphate buffered saline (PBS) and stored. Skin scrapings were obtained from healthy skin or lesions of the medial pinna using sterile needles or surgical blades, and then stored in 0.2 ml of PBS. Saliva samples were collected using a sterile swab and stored in 0.5 ml of saline solution, then centrifuged at 10,000 rpm for 10 minutes and the supernatant retained, and stored at -20°C; however, pilot saliva IgA ELISA yielded no results and sampling was discontinued.

In June and July 2019, 30 additional dogs were sampled in areas of Puerto Iguazú, also selected by worst-scenario sampling. The owners were interviewed, and the dogs examined for clinical signs, as above. For these 30 dogs, dental broach, dental brush, and LNA samples were taken from each animal. A barbed 25 mm dental broach (Billericay Dental Supply, UK) was used to take a biopsy from the skin of the ear pinna, using the method employed in diagnosis of HCL [25]. Two broach samples were taken from each dog, one placed into 2 ml of culture medium (αMEM, M0644, Sigma-Aldrich, UK), and the other into cell lysis buffer (10 mM TrisCl, 1 mM EDTA, 100 mM NaCl and 1% SDS). Dental brushes (DenTek, UK) were brushed onto skin and stored in cell lysis buffer. Fine needle aspirate of the popliteal lymph node was performed, and the samples stored in cell lysis buffer at room temperature.

### Microscopy, serological and molecular tests

**Fig 2:**
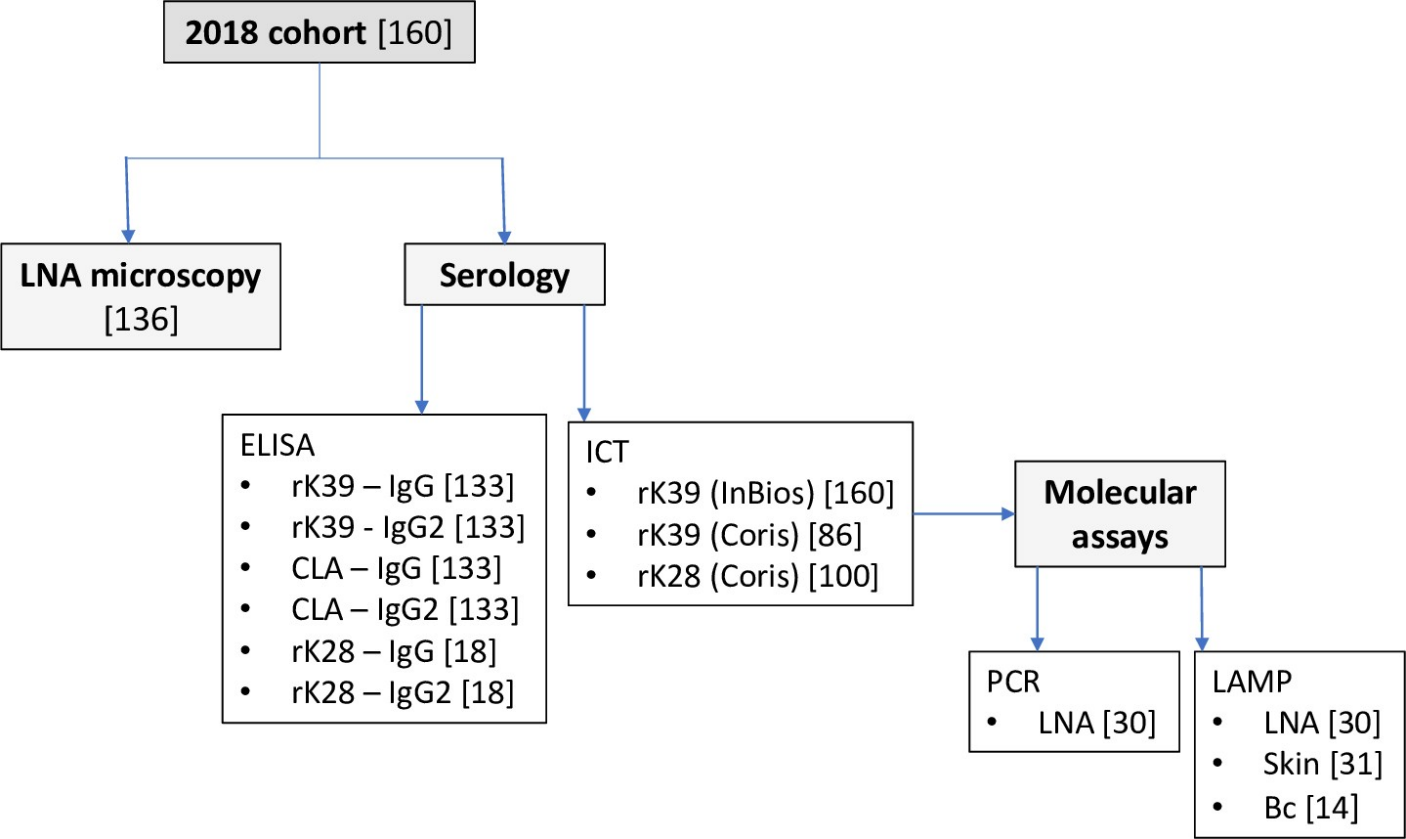
Summary of microscopy, serology and molecular tests applied to the 2018 cohort of 160 dogs (number of dogs to which each test was applied shown in square brackets). Samples were selected for LNA-PCR and LNA-LAMP based on ICT results. Serology and PCR-RFLP with skin broach and LNA samples were also applied to the additional 30 dogs sampled in 2019, and those seropositive for rK28 ICT but negative with rK39 ICT were tested serologically for exposure to six other pathogens (Methods). Bc, buffy coat; CLA, crude lysate antigen; ICT immunochromatographic test.

The diagnostic tests performed are shown in Fig 2. These included: LNA light microscopy; three rapid immunochromatographic tests (ICTs) on serum, enzyme-linked immunosorbent assay (ELISAs) for total IgG and IgG subclass on serum, using antigens rK39, rK28 and soluble cell lysate antigen (CLA); loop-mediated isothermal amplification (LAMP) of DNA on buffy coat, skin scrape and LNA; polymerase chain reaction (PCR) on LNA; PCR-restriction fragment length polymorphism (PCR-RFLP) on skin broach and LNA samples. Due to availability of reagents and kits, and sample quality, each test was not performed on every dog.

#### LNA light microscopy

Smears were examined from LNA samples fixed with methanol and stained with Giemsa at 1000x magnification for the presence of *Leishmania* amastigotes.

#### Antigen sources for use in prototype ICTs and ELISAs

Recombinant proteins rK28 (CTK Biotech, USA) and rK39 (RAG0061, Rekom Biotech, Spain) were obtained commercially. *Leishmania donovani* whole-cell promastigote lysate was derived from the culture-adapted strain MHOM/IN/80/DD8, grown in αMEM with supplements as described previously [26]; cell lysate was prepared as described previously [27].

#### ICT

Three lateral flow ICTs were applied with the serum samples: the Kalazar Detect Canine Rapid Test (Inbios Inc., USA, with protein A detection of IgG) employing rK39, used as per manufacturer’s instructions, and two prototypes (both with protein G detection of IgG) with rK39 (VL Sero K-SeT rK39) or with rK28 (VL Sero K-SeT rK28) from Coris BioConcept, Belgium. rK39 is comprised of several repeat regions of the *L*. *infantum* kinesin protein; in contrast the synthetic fusion protein rK28 has two kinesin repeat regions flanked by repeat regions of the HASPB protein, all derived from *L*. *donovani* [28]. These two novel RDTs were composed of a nitrocellulose strip sensitized with recombinant antigen (rK28 or rK39, sourced as above) and with the Protein G conjugated to colloidal gold, housed within a plastic cassette with a buffer application well and a test/reading window. Serum/plasma at volumes of 3.5 μl was pipetted onto the sample application zone in the test/reading window, and then 120 μl of buffer solution was dispensed into the buffer application well. After 15 minutes, a test was deemed valid if a red control band was present in line with the “C” on the cassette and was deemed positive if a second band was present in line with the “T”. If no band was visible at the “T”, then the test was deemed negative.

#### ELISA

ELISAs were optimised by checkerboard dilution of serum and secondary conjugates. ELISAs were then performed using a CLA of *L*. *donovani* promastigote strain MHOM/IN/80/DD8, and rK39 and rK28 antigens as described above and in Fig 2. Flat-bottom 96-well ELISA plates (735–0465, VWR, UK) were coated separately with 2 μg/ml of CLA or 0.3 μg/ml of rK28 or rK39, diluted in coating buffer (15 mM Na_2_CO_3_, 34 mM NaHCO_3_, pH 9.6) at 100 μl/well and incubated with an adhesive cover at 4°C overnight. Following three washes with PBS/0.05% Tween 20 (PBST), 200 μl/well of blocking buffer (PBS/2% skimmed milk powder, Premier Foods, UK) was applied and incubated for 2 hours at 37°C. Following three PBST washes, 100 μl/well of canine serum diluted 1:200 in PBST/2% milk (PBSTM) was added. After incubation at 37°C for 1 hour and six washes in PBST, 100 μl/well of one of the following HRP-conjugated secondary antibodies diluted at 1:2,000 in PBSTM was added: goat anti-dog IgG1 (A40-120P, Bethyl Laboratories, USA); sheep anti-dog IgG2 (A40-121P, Bethyl Laboratories); rabbit anti-dog IgG (304-035-003; Jackson Immunoresearch, USA). Following incubation at 37°C for 1 hour and six PBST washes, reactions were developed using 100 μl/well of ABTS substrate (50-62-00, SeraCare, USA) and stopped with 50 μl of 2M H_2_SO_4_; absorbance values were determined at a wavelength of 405 nm. Samples were assayed on duplicate plates simultaneously. Positive controls were obtained from local archived positive sera. Negative controls were from non-endemic dogs without *Leishmania* or *Trypanosoma cruzi* infection. Cut-off values were set by defining the mean value from serum of uninfected dogs plus three standard deviations.

#### PCR

DNA extraction from the LNA samples (Fig 2) was first carried out using the DNA Puriprep-S Kit (Inbio Highway, Argentina) as per the manufacturer’s instructions with minor modification. *Leishmania* spp. heat shock protein 70 gene (HSP-70) DNA was amplified using PCR-N primers as described previously [29]. The reactions were carried out in a final volume of 25 μl containing 5 μl of DNA template, 0.5 μM of primers F25 (ggacgccggcacgattkct) and R617 (cgaagaagtccgatacgaggga) and 1x GoTaq Green Master Mix, (Promega, USA). Amplification conditions were: 95°C for 5 min; 40 cycles at 95°C for 40 sec, 65°C for 1 min, 72°C for 1 min; 72°C for 10 minutes. Reaction products were electrophoresed on 2% agarose gel stained with Sybr Green (Invitrogen, USA).

#### PCR-RFLP

For the 30 dogs sampled in 2019, PCR-RFLP was carried out with broach and LNA samples; RFLP could not be applied to brush samples, because they did not yield PCR products. Samples were placed in 2 ml of cell lysis buffer solution prior to DNA extraction using the QIAamp DNA Mini Kit (51304: Qiagen, UK). Briefly, 1/10th volume of Proteinase K and an equal volume of buffer AL were added to either 400 μl or 1 ml of lysis buffer containing the samples. After incubation at 56°C for 10 minutes, absolute ethanol equal to the sample volume was added, and thereafter the protocol continued according to manufacturer’s instructions. DNA was eluted in 200 μl buffer AE.

Extracted DNA were used in PCR for species identification using either the ITS1 region [30], or HSP-70/PCR-N [29]. Reactions consisted of 1 U BioTaq polymerase and supplied NH4 reaction buffer (Bioline, UK), 1.5 mM (for ITS1) or 2.5 mM (for HSP-70) MgCl_2_, 200 μM dNTPs, 0.5 μM of ITS1 primers LITSR (ctggatcattttccgatg) and L5.8s (tgataccacttatcgcactt), or HSP-70/PCR-N primers described above. In addition, PCR-N reactions contained 1 x High GC Enhancer (New England Biolabs, UK). Amplification conditions for ITS1 were: 1 cycle of 94°C for 2 mins; 33 cycles of 94°C for 30 secs, 53°C for 30 secs, 72°C for 1 min; and 1 cycle of 72°C for 10 mins. Amplification conditions for HSP70/PCR-N were: 1 cycle of 95°C for 5 mins; 35 cycles of 94°C for 40 secs, 61°C for 1 min, 72°C for 1 min, and 1 cycle of 72°C for 10 mins.

Following the ITS1 PCR, half of the reaction was digested with *Hae*III (R0108S, New England Biolabs) in supplied 1x buffer, and then electrophoresed on 3% or 3.5% agarose gel. Additionally, ITS1 amplicons from two of the skin samples were subcloned into pGEM-T easy vector (A1360: Promega, UK), and DNA sequences from each of 12 resultant clones were analysed. For PCR-N, the amplicon was electrophoresed directly on 1% gel.

#### LAMP

LAMP was performed on skin scrapes (skin LAMP), buffy coat (bc LAMP) and LNA (LNA LAMP), for the 2018 cohort of dogs (Fig 2). First, DNA was extracted from LNA samples as described above, and using DNeasy Blood & Tissue Kit (Qiagen, Germany) for skin scrapes and buffy coat suspensions. The Loopamp *Leishmania* Detection kit (Eiken Chemical, Tokyo, Japan), which amplifies 18S rDNA (nuclear) and kinetoplastid DNA sequences, was used according to manufacturer’s instructions. Briefly, 30 μl of a 1:5 dilution of DNA in water was added to the reaction tube containing the lyophilised reagents, which was then inverted to reconstitute the dried reagent in the cap. Samples were incubated at 65°C for 40 minutes, and then the reaction was terminated by heating at 95°C for 2 mins. Positive samples generate a turbid green colour under UV light at 350–370 nm using LE Ultra Violet LED Flashlight/Blacklight Torch (Lighting Ever, Birmingham, UK) or UVGL-58 Lamp (UVP, California, USA).

#### Tests for other pathogens

Serum samples from the 2019 group of 30 dogs that were positive with the prototype rK28 ICT but negative with the rK39 ICT were tested for other vector-borne pathogens, to investigate the possibility that higher positive results with the rK28 ICT were due to cross-reaction with other infections. The samples were subjected to a serology-based RDT (SNAP 4Dx Plus Test, Idexx, UK) which screens for *Dirofilaria immitis*, *Ehrlichia canis*, *Ehrlichia ewingii*, *Anaplasma phagocytophilum*, *Anaplasma platys* and *Borrelia burgdoferi* sensu lato. The tests were used as per the manufacturer’s instructions.

### Statistical analysis

Agreement between tests for the 160 dogs (Fig 2) was calculated using Fisher’s exact test, and Cohen’s kappa coefficient (for which values of 1.00–0.81 were interpreted as excellent agreement, 0.80–0.61 good, 0.60–0.41 moderate, 0.40–0.21 weak, and 0.20–0.00 negligible). To measure association between IgG1 and IgG2, and disease severity, Pearson’s correlation coefficient and one-way analysis of variance (ANOVA) were performed. *P* values < 0.05 were considered statistically significant.

## Results

### Population characteristics and clinical signs

**Fig 3:**
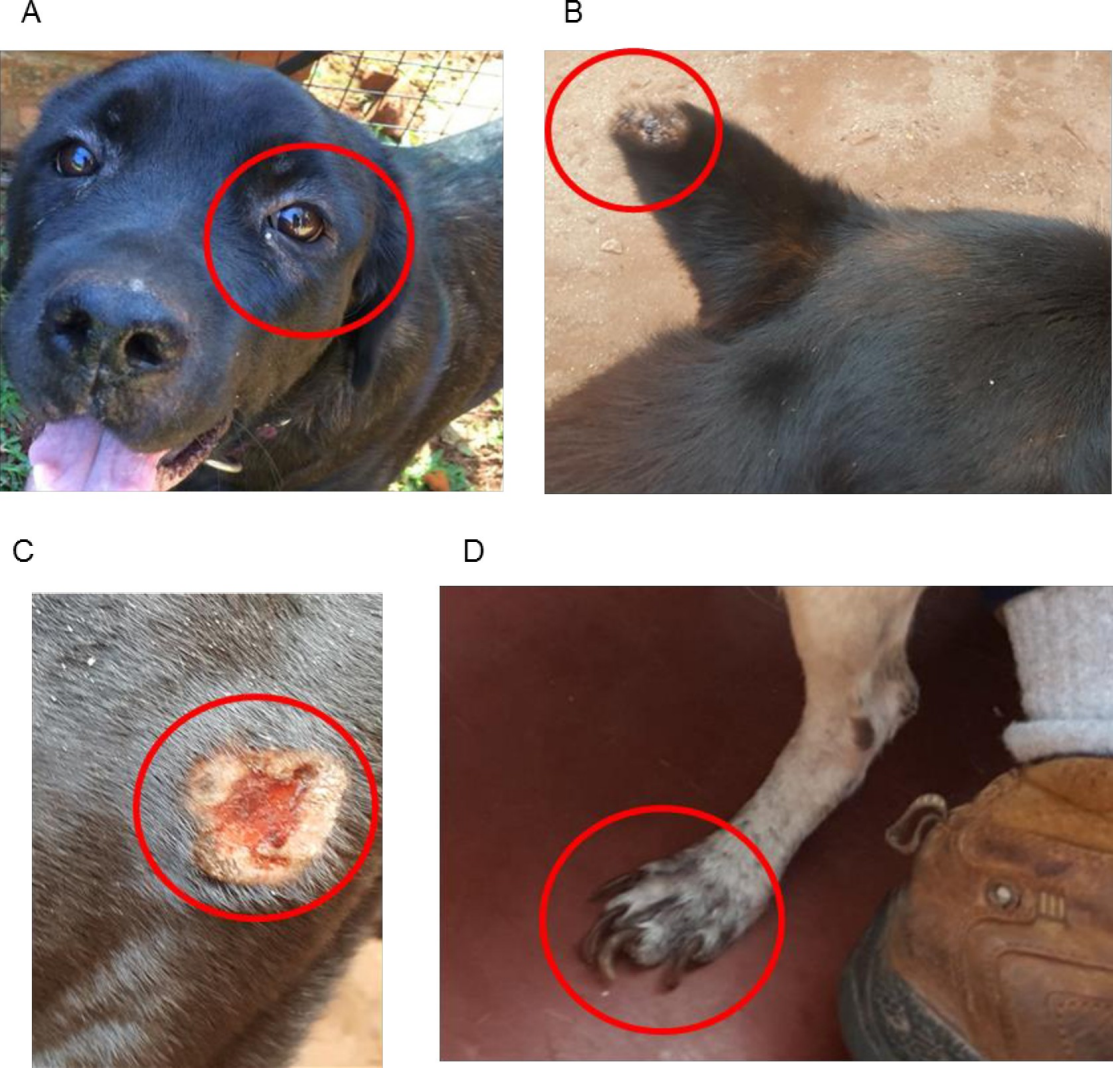
Examples of observed clinical signs of canine visceral leishmaniasis: A. localised alopecia, B. ear chancre, C. chancre in the gluteal region, D. onychogryphosis.

Demographic data available for the 160 dogs enrolled in 2018 and for the additional 30 dogs in 2019 are summarised in Table 1. Frequency of each clinical sign for the 160 dogs is also shown. Overall, 52/160 (32.5%) of the dogs were asymptomatic, 78/160 (48.75%) oligosymptomatic, and 30/160 (18.75%) polysymptomatic. Fig 3 shows examples of some of the clinical features observed.

**Table 1:** Demographic and clinical data for 2018 (160 dogs), and where available additionally for 2019 (30 dogs).

	Frequency	%
Sex, male	96/190	50.5
Age (years)		
0.5–1	10/190	5.3
1–10	153/190	80.6
>10	16/160	10.0
Unknown	6/160	3.8
Mixed breed	173/190	91.1
Clinical signs		
Alopecia	60/160	39.4
Dermatitis	17/160	11.4
Chancre	27/160	16.9
Conjunctivitis	5/160	3.1
Onychogryphosis	12/160	7.5
Lymphadenopathy	69/160	43.1
Weight loss	10/160	6.3

### Lymph node microscopy

Twenty one of 136 (15.4%) dogs enrolled in 2018 examined by LNA microscopy were positive for the presence of *Leishmania* amastigotes.

### Serology

#### ICTs and ELISA

Of dogs enrolled in 2018, 30/160 (18.8%) of serum samples were positive with the commercial rK39 test (InBios) and 27/86 (31.4%) with the rK39 prototype (Coris). The rK28 prototype (Coris) had a significantly greater apparent sensitivity with 61/100 (61%) positive. Table 2 shows the agreement between the serological tests and LNA microscopy by Cohen’s kappa coefficient. The commercial rK39 test had good concordance with the prototype rK39 test (κ = 0.739), moderate concordance with microscopy (0.463), and only weak concordance with the prototype rK28 test (0.352), explicable by the substantially higher seropositivity of the prototype rK28 test.

**Table 2:** Estimated concordance between diagnostic tests by Cohen’s kappa coefficient (95% confidence intervals). *p<0.01, **p<0.05 by t-test.

	rK39-ICT (Coris) n = 86	rK28-ICT (Coris) n = 100	ELISA (n = 133)				LNA-microscopy n = 136
			rK39-IgG	CLA-IgG	rK39-IgG2	CLA-IgG2	
rK39-ICT (InBios) N = 160	0.739* (0.578, 0.901)	0.352* (0.182, 0.521)	0.780* (0.649, 0.912)	0.398* (0.234, 0.563)	0.339* (0.176, 0.502)	0.611* (0.454, 0.768)	0.463* (0.252, 0.675)
rK39-ICT (Coris)	–	0.443* (0.259, 0.627)	0.681* (0.505, 0.857)	0.481* (0.287, 0.674)	0.393* (0.192, 0.593)	0.639* (0.457, 0.820)	0.437** (0.174, 0.699)
rK28-ICT (Coris)	–	–	0.310* (0.138, 0.483)	0.302* (0.119, 0.485)	0.217* (0.029 0.405)	0.320* (0.146, 0.494)	0.253* (0.067, 0.440)
rK39-IgG	–	–	–	0.500* (0.346, 0.654)	0.433* (0.277, 0.588)	0.698* (0.559, 0.837)	0.402** (0.172, 0.633)
CLA-IgG	–	–	–	–	0.426* (0.272, 0.581)	0.668* (0.538, 0.799)	0.132 (-0.065,0.328,)
rK39-IgG2	–	–	–	–	–	0.497* (0.348, 0.646)	0.094 (-0.105, 0.291)
CLA-IgG2	–	–	–	–	–	–	0.238** (0.01, 0.467)

Total IgG and IgG2 levels were assessed further, by anti-rK39 and anti-CLA ELISAs with 133 dogs. The number of dogs positive on each ELISA were: rK39-IgG 31/133 (23.3%); CLA-IgG 58/133 (43.6%), rK39-IgG2 64/133 (48.1%), CLA-IgG2 41/133 (30.8%). Thus, IgG2 was more sensitive than IgG with rK39, and less sensitive than IgG with CLA. However, in all clinical groups IgG2 levels were correlated with total IgG in both rK39 ELISA (r = 0.912, 95% CI: 0.878, 0.937, *p*<0.001) and in CLA ELISA (r = 0.943, 95% CI: 0.920, 0.959, *p*<0.001).

For 34 dogs with a positive PCR and/or LAMP result (see below), the average positivity rate was 49.3% across all four ELISAs, highest with rK39-IgG ELISA (58.8%) and lowest on rK39-IgG2 ELISA (38.2%). Eighteen canine sera from Argentina plus four healthy control sera from non-endemic dogs were tested with anti-rK28 IgG and anti-rK28 IgG2 ELISAs. There was 100% agreement between both ELISAs, with 16/18 dogs (88.9%) positive on both.

### Molecular tests: PCR and LAMP

#### LNA, skin and buffy coat

30 dogs of the 2018 cohort were selected for LNA PCR, and for LNA, skin and buffy coat LAMP. By LNA-PCR using PCR-N primers, 3/30 (10%) were positive and by LNA LAMP 22/30 (73%) positive when visualised with UV light.

**Fig 4:**
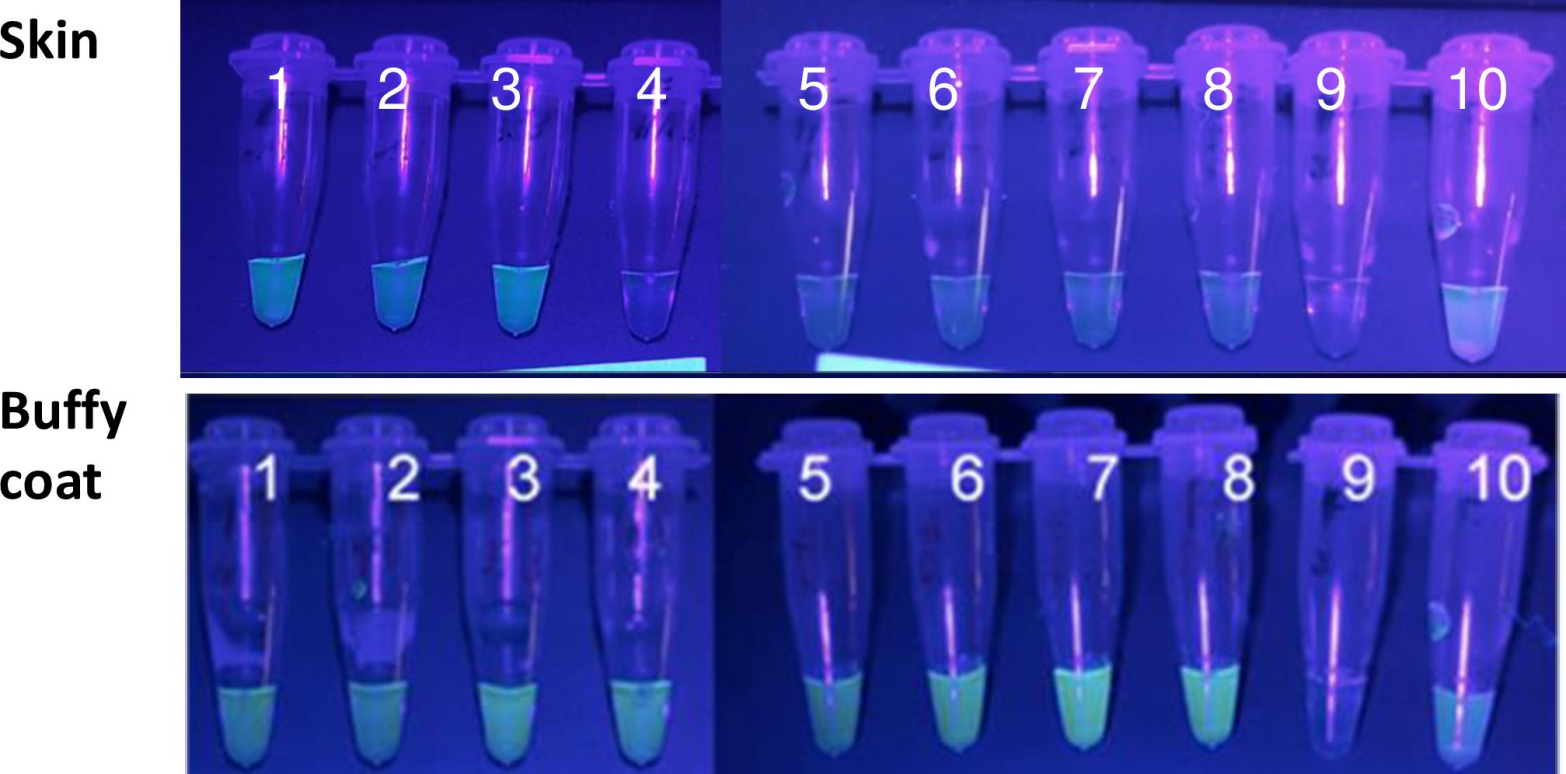
Skin-LAMP and buffy coat LAMP under UV light show higher sensitivity of buffy coat LAMP (samples 1–8); 9, distilled water (no template); 10, positive control.

To assess the validity of skin scrape samples for use in LAMP, samples from a group of 31 dogs with variable clinical signs were tested. 11/31 (35.5%) were LAMP positive under UV light. A higher proportion with skin lesions (7/9, 77.8%) were positive than those without lesions (7/22, 31.8%; *p*<0.05). Seven dogs with negative results on skin LAMP, and 7 with positive results, were subjected to a reference test of buffy coat LAMP, and all were positive, indicating that sensitivity of skin LAMP was lower than that of buffy coat LAMP (Fig 4).

### Assessing cross-reactivity

The high seropositivity rate among rK28 RDTs suggested that this was the most sensitive of the serological tests. Twenty four of 28 dogs that were positive with the rK28 ICT were negative with the commercial rK39 ICT. To assess whether the high seropositivity of rK28 might be due to cross reactivity with other pathogens, the 24 dogs with inconsistent results were tested by LNA PCR and LNA LAMP. Of the 24 negatives with the rK39 ICT, with corresponding DNA samples eighteen were positive by LAMP, of which 2 were positive by PCR, and thus the rK28 results were not explicable by false positivity.

We also tested 10 positives by rK28 that were negative with one or both rK39 ICTs using the SNAP 4DX test to detect serological cross reactions with other canine infections. A single sample was serologically positive for *A*. *phagocytophilum/A*. *platys* suggesting prior exposure/infection but without confirmation of current co-infection. Thus, rK28 seropositives indicated presence of *Leishmania* infection, and, with the possible exception of this one sample, no cross reactions with any of the six other canine infectious diseases tested.

### Disease severity: correlation of diagnostic profile and clinical signs

**Fig 5:**
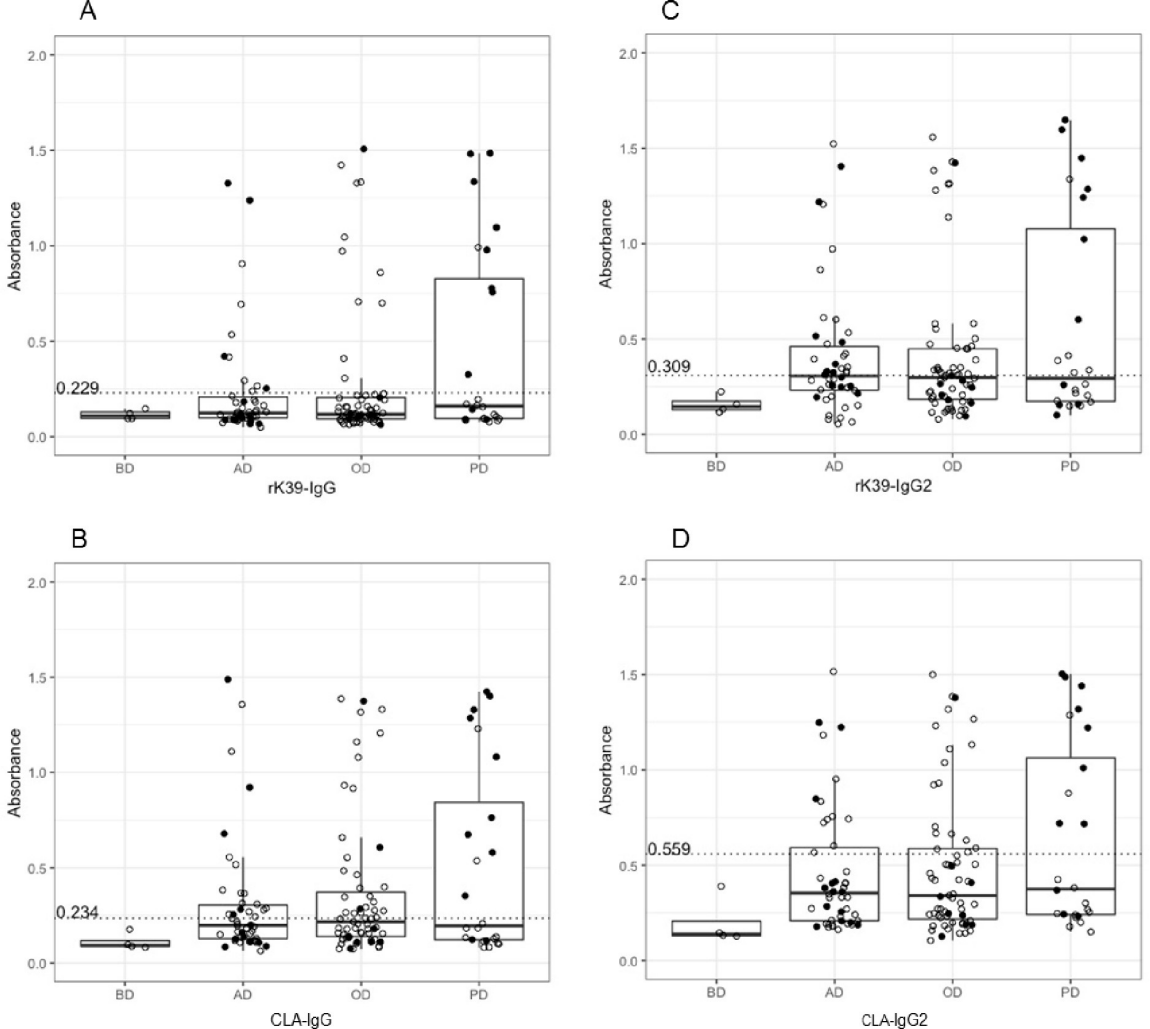
Anti-rK39 and anti-CLA antibody levels in serum suggest elevation of rK39 antibody titres (*p* = 0.045) in polysymptomatic disease. (A) rK39-IgG, (B) CLA-IgG, (C) rK39-IgG2 and (D) CLA-IgG2 in 133 domestic dogs and four dogs from a non-endemic area. The 133 dogs were classified by clinical signs: BD, dogs from a non-endemic area; AD, asymptomatic; OD, oligosymptomatic; PD, polysymptomatic. Black dots show dogs detected as *Leishmania*-positive by molecular assays.

A higher level of total IgG by anti-rK39 ELISA was observed with dogs recorded (Table 1) as having increasing number of clinical signs (*p* = 0.045), analysed according to asymptomatic, oligosymptomatic or polysymptomatic status. However, there was no statistically significant association between this limited classification of clinical signs and antibody level with anti-rK39 IgG2, anti-CLA IgG, anti-CLA IgG2, anti-rK28 IgG or anti-rK28 IgG2 ELISAs (Fig 5).

### Co-endemicity of canine *L*. *infantum* and *L*. *braziliensis* infections

**Fig 6:**
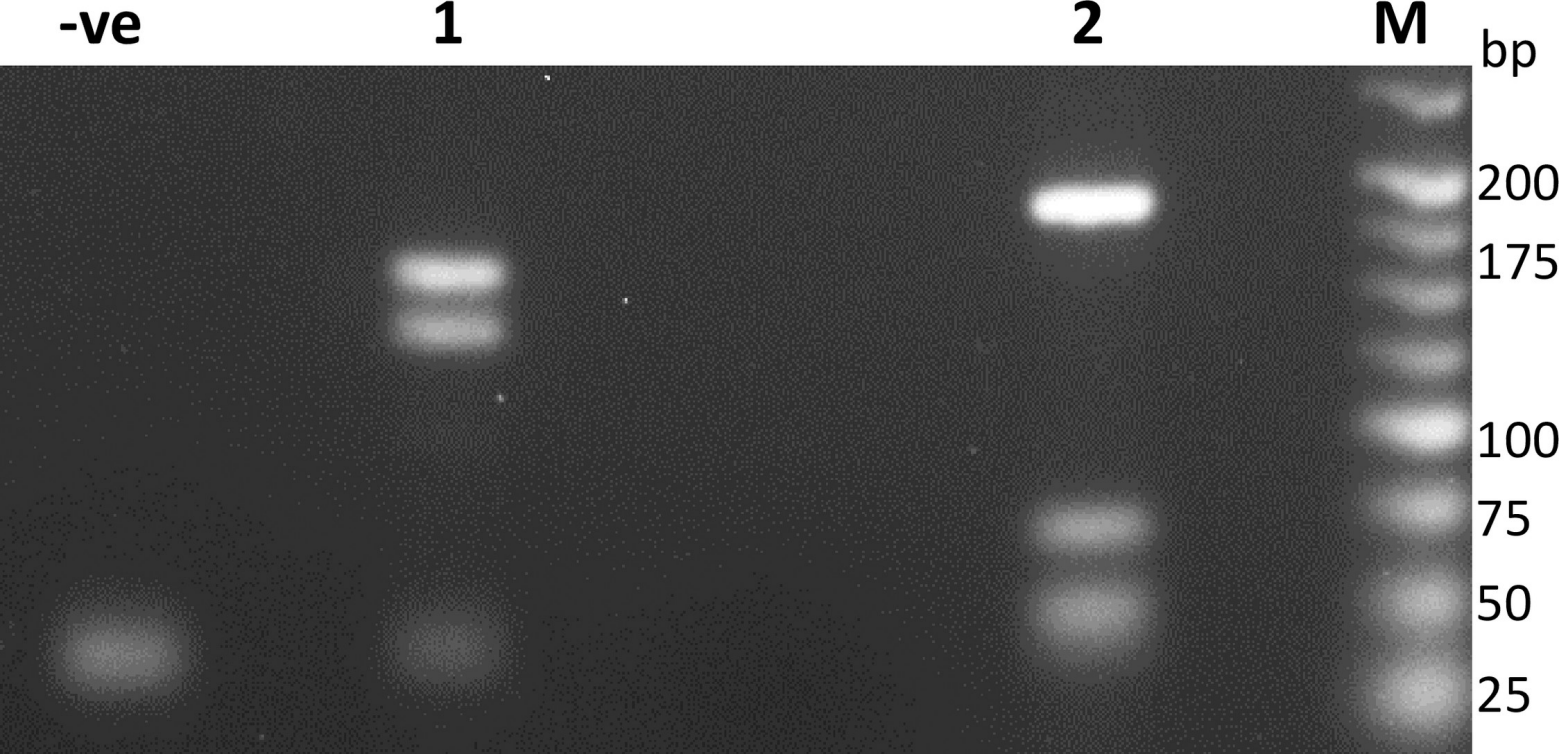
PCR-RFLP using ITS1/*Hae*III reveals the presence of canine leishmaniasis compatible with *Viannia* infection (see Discussion) (Lane 1, broach sample) in Puerto Iguazú, as well as CVL due to *L*. *infantum* (Lane 2, lymph node aspirate); -ve, negative control; M, size markers.

Identification of *Leishmania* species was performed via ITS1/*Hae*III PCR-RFLP. This analysis identified *L*. *infantum* as the agent of visceral leishmaniasis in the dogs. However, an ITS1/*Hae*III PCR-RFLP pattern consistent with *L*. *(Viannia) braziliensis* was identified with samples from the ear lesions of two dogs (Fig 6). Amplicons from the two dogs were cloned into plasmids, and, for each of the two dogs, 12 plasmid colonies were sequenced; GenBank accession numbers MW683339 and MW683340 are the derived representative sequences. Consistent with the skin lesion clinical presentations, BLAST comparisons of the ITS-1 sequences indicated *L*. *braziliensis*, with percentage identity score of 99.35% and Max score of 551. Percentage identity and Max scores against *L*. *guyanensis* were 99.01% and 542, respectively. PCR-N primers did not produce amplicons from these two dogs, hence HSP70 sequences could not be used as a means of species identification.

S1 Table consolidates the results of the 2018 and 2019 dogs in this study.

## Discussion

Serological and parasitological profiling of asymptomatic, oligosymptomatic and polysymptomatic dogs revealed notable differences in sensitivities of the diagnostic tests.

The low sensitivity of reference tests may underestimate detection of true positives/sensitivity of comparator tests [22]. Many publications have also described the need for the use of more than one test to diagnose CVL, as serological tests cannot differentiate between immune and infectious dogs [22]. There is evidence that the performance of various RDTs for leishmaniasis diagnosis, including Kalazar-Detect (Inbios) and other rK39 and rK28 RDTs may vary according to severity of disease [11,31,32].

As far as we are aware, this is the first report of LAMP for diagnosis of CVL in Latin America. The Eiken LAMP kit, first reported by Adams et al. [33] and used subsequently for HCL and HVL [34–37], has been shown to be effective for diagnosis of leishmaniasis. The higher positivity rate with LAMP than PCR may be explicable by the Eiken LAMP method targeting highly repetitive sequences. However, two in-house, non-commercial LAMP techniques described for diagnosis of CVL [38,39] were not used. More research on existing and new sampling methods is needed. In our diagnostic comparisons the dental broaches were inserted into tissue to obtain biopsies. Broaches have been used successfully for HCL diagnosis [40].

Detection of *Leishmania* DNA by LAMP and PCR supported the fidelity of the rK28 ICT tests. Nor did cross-reactivity with the pathogens tested on the SNAP 4DX explain the higher number of positives for the rK28 ICT versus both rK39 ICTs; with the exception of a single sample positive *for Anaplasma* spp., all SNAP 4DX tests were negative. It is possible that there may be cross-reactions with other infections that we have not tested, such as *Babesia canis*, which has been reported to cross-react with another rK28-based RDT [41].

It is known that a cell-mediated immune response is associated with protection against symptomatic leishmaniasis [42] whilst high antibody titres are associated with more severe disease in both dogs and humans [11,43,44]. This is reflected in our finding that total IgG was higher in anti-rK39 ELISA amongst dogs with more clinical signs, although with our cohort of dogs this association was not demonstrable for the anti-CLA or anti-rK28 ELISAs. Whilst we did not find a correlation between IgG or IgG2 level and disease severity, more research is needed to identify reliably deployable biomarkers of clinical status. There is conflicting evidence regarding the link between canine anti-*Leishmania* IgG subclass profile and disease state, particularly whether IgG1 or IgG2 is associated with symptomatic disease [45–50]. This may be due to lack of specificity of the commercially available polyclonal antisera used in such studies, including in this research [51]. In humans IgG1 has been shown to be an important marker of relapse [52], and it has been shown canine IgG2 may be analogous to human IgG1 [53]. Furthermore, to explore biomarkers a more comprehensive classification of clinical status is required, not only asymptomatic, oligosymptomatic and polysymptomatic, because the characteristics of the lesions may be associated with parasite loads and influence the immune response.

A key area for development of improved diagnostics for CVL is the field of differential diagnosis of varying infection states. It is well established that infectiousness varies between dogs [54]; this is analogous to the heterogeneous spread of human visceral leishmaniasis (HVL) from HVL and post-kala azar dermal leishmaniasis cases due to high variation in infectiousness between cases [55]. If ‘super-spreader’ dogs that are particularly infectious to sand flies could be identified by a highly specific test, this could be used to inform selective culling, which is likely to be more effective at reducing human infection rates than blanket culling of dogs, as well as more humane [56]. Another pressing issue, given the increasing availability of CVL vaccines [57], is rapid differentiation of vaccinated from naturally exposed dogs. This is reported to be feasible via ELISA, with rK28 having lower cross-reactivity than other antigens with the Leish-tec vaccine specifically [17], but rapid point of care tests are needed for use in the field.

Using the HSP70 PCR-N PCR primers, Fernández et al. [58] reported the presence of *L*. (*V*.) *braziliensis* in one individual each of *Akodon* sp. and *Euryoryzomys russatus* rodents surveyed from south of Puerto Iguazú city. Here, using ITS1 PCR primers, in addition to *L*. *infantum* we identified *Leishmania* (*Viannia*) spp. from skin lesions of two dogs. The HSP70 PCR-N primers with the two *L*. (*Viannia)* samples did not produce amplicons to enable unequivocal identification of the species. However, the ITS1/*Hae*III PCR-RFLP profile and the Blast comparisons of ITS DNA sequences identified *L*. *braziliensis*. This identification is supported by the local epidemiology: *L*. *braziliensis* is a cause of HCL in Argentina [3], isolated previously from dogs [59]. A survey of vector distribution in Puerto Iguazú found the most abundant sand fly species to be *Lutzomyia whitmani*, a primary vector of *L*. *braziliensis*, followed by *Lu*. *migonei*, a secondary vector [60]. The known susceptibility and widespread emergence of suburban *L*. *braziliensis* in dogs [61–63] supports our discovery of *L*. *braziliensis*. This result encourages further research on the identification of animal reservoirs, both sylvatic and domestic, of *L*. *braziliensis* [64], and highlights the need to consider measures to prevent its spread in canine and human populations.

## Limitations

A limitation of this study is the lack of a gold standard test for definitive diagnosis of CVL; LNA microscopy is considered a gold standard but sensitivity is low. Due to limited availability of tests, not all tests could be applied to every dog. LAMP was only performed on the LNA, skin and buffy coat samples of 30 dogs of 2018. As discussed, IgG subclass ELISA is limited by the lack of certain subclass specificity of commercially available antisera, so relationship between disease status and IgG1 or IgG2 may require precision monoclonal antisera. LAMP tests require visual judgement and observer blinding. This work focused on owned dogs, whilst stray dog populations are also an important reservoir of *L*. *infantum* [8].

## Conclusions

Comparisons of methods of diagnosis for CVL have shown that, for the range of asymptomatic, oligosymptomatic and polysymptomatic dogs, serology is far more sensitive than parasitological methods. Of the serological methods, the rK28 prototype was significantly more sensitive and has great promise as a point-of care RDT. PCR and LAMP detection of DNA, and serology to eliminate presence of multiple other pathogens, indicated that the efficacy of the rK28 RDTs was probably not attributable to false positives, although this requires follow up with a wider range of canine infections. As far as we are aware, we have described the first application of LAMP to CVL in Latin America, with encouraging results; however, cost and accessibility to the one proven commercial LAMP assay prohibits its wide deployment for surveillance and control of CVL. In applying molecular methods for diagnosis, we detected evidence of local presence of canine leishmaniasis due to *L*. *braziliensis*, requiring further research and vigilance, although impact of dogs on transmission of infection is uncertain. Clearly, more research is required to enhance understanding of CVL, optimise rapid diagnosis, produce a canine surveillance algorithm, and to foresee and control the widening spread of this potentially devastating neglected veterinary and public health problem.

## Supporting information

S1 Table Consolidated results of dogs in the 2018 and 2019 studies. (XLSX)
